# Trends in age-specific varicella incidences following the introduction of the general recommendation for varicella immunization in Germany, 2006–2022

**DOI:** 10.1186/s12889-023-17098-1

**Published:** 2023-11-08

**Authors:** Felix Moek, Anette Siedler

**Affiliations:** 1 Department of Infectious Disease Epidemiology, Postgraduate Training for Applied Epidemiology (PAE), Robert Koch-Institute, Berlin, Germany; 2 European Programme for Intervention Epidemiology Training (EPIET), European Centre for Disease Prevention and Control (ECDC), Stockholm, Sweden; 3 Department of Infectious Disease Epidemiology, Robert Koch-Institute, Berlin, Germany

**Keywords:** Chicken pox, Varicella-zoster virus, Vaccine-preventable diseases, Varicella vaccine

## Abstract

**Background:**

In Germany, general childhood varicella vaccination has been recommended since 2004. A feared effect of low vaccination coverage is a possible shift in incidence from children to teenagers and young adults who are at higher risk of severe outcomes. If true, this shift would possibly necessitate changes to the national immunization strategy.

We aimed to evaluate the effects of the general vaccination recommendation on age-specific varicella incidences in Germany in general and examine specifically whether a shift from children to teenagers (15 to 19 years) has occurred.

**Methods:**

Trends in age-specific incidences were evaluated using triangulation with the following datasets: national mandatory notification data (*N)* (2014–2022), billing data of the statutory health insurance associations (*I*) (2009–2017) and data from a doctor's sentinel system (*S*) (2006–2017). Similar clinical case definitions were used in *N* and *S*, while *I* used ICD-10-codes. Age groups were stratified as available in all three systems. Incidences per year were calculated based on the total population (*N*), the number of statutory health insured (*I)*, and extrapolated from *S* to the total population.

**Results:**

During all years of observation, age-specific incidences have dropped significantly across all age-groups for *S* und *I.* The age groups (under 10 years) with initially highest incidences were the ones with the strongest reductions (under 1 year: -90%, 1–4 years: -95.5%, 5–9 years: -89.2% for *S*; -67.7%, -78%, -79.3% for *I*). A single 53.1% increase in the low incidence in *S* among 15–19-year olds observed in 2017 compared to 2016 could not be confirmed in *N* or *I*. Increases in incidences during the first two years of *N* are probably due to improved notification behaviour over these years. In 2019, all age-specific incidences increased (*N*), with 15 to 19-year olds showing the highest relative increase (28.2%).

**Conclusions:**

Since the introduction of the general vaccine recommendation against varicella, incidences across all age-groups have declined significantly. Available data indicate no evidence for a shift in disease incidence to older age groups. Every incidence increase beyond childhood age should however be followed up closely. So far, children and adolescents have both benefitted from the current vaccination strategy.

## Background

The clinical disease of varicella is the consequence of a primary infection with the varicella-zoster virus (VZV). In susceptible populations, it can cause significant morbidity and thus puts a considerable burden on countries´ health and economic systems. In countries of the western hemisphere young children under 10 years are mainly affected. However, an effective vaccination is available to limit this burden.

In Germany, the Standing Committee on Vaccination (STIKO) made a general recommendation for the vaccination of children in 2004, thus responding to several hundred thousand cases of varicella that occurred every year [1]. Five years later, the recommendation was further expanded and then envisaged two vaccinations, the first during the age of 11–14 months, the second during 15–23 months, nowadays at 11 and 15 months. Germany is one of the few countries worldwide that have added a general recommendation for VZV immunization to their national immunization schedule. Vaccination is voluntary and usually offered by the treating pediatrician or general practitioner. Costs for both vaccination and treatment are covered by the individual`s health insurance.” Vaccination coverage has reached 88.9% (85.1%) for the first (second) dose for 4 to 7-year-old children at school entry examination in 2020 [2].

One of the feared negative effects of VZV immunization in childhood—which might discourage other countries from adopting it—is the possible risk of a shift of disease burden from younger children towards teenagers and young adults [1]. This would be concerning as older individuals have a higher risk of a complicated disease course. This fear is based on the following reasoning: If coverage is not high enough to successfully limit virus circulation and grant herd immunity, the proportion of susceptible persons is growing and their first virus exposure may happen later in life. According to modelling results the threshold for herd immunity was calculated as >  = 80% population immunity for Germany [3].

Hitherto, no evaluation of the long-term effects of the general recommendation for VZV immunization on age-specific varicella incidences in Germany has been performed. We therefore aimed to:

1. describe trends in age-specific varicella incidences in Germany in general and
2. examine specifically whether a shift in absolute varicella incidence from younger ages towards teenagers (aged 15 to 19 years) has occurred,

following the introduction of the general recommendation for varicella immunization.

The results of this study are relevant as the aforementioned, possible rise in incidence and morbidity in teenagers and young adults might require either a modification of the current varicella vaccination schedule or increased efforts to improve the vaccine uptake according to the existing schedule.

## Methods

### Study design

This was a retrospective, observational study on the age-specific incidences of varicella in Germany from 2006 to 2022, with a specific focus on the age group of teenagers (15–19 years old).

### Data sources

The analysis was done using a triangulation approach with the following three datasets:

1. A sentinel system for varicella, with data from 2006 to 2017 (*S*)
2. Physicians’ claims data on statutory health insured persons, with data 2009 to 2017 (*I*)
3. The countrywide statutory notification system, with data from 2014 to 2022 (*N*)

All three datasets comprise data on varicella incidence on a nationwide scale. The characteristics of each data source will be described in the following.

#### Varicella sentinel data (*S*)

After the addition of the varicella vaccine to the routine immunization scheme in Germany in 2004, there was a need to monitor the impact of the vaccination. As varicella was not a generally notifiable disease at that time, RKI decided to use a sentinel surveillance system to monitor varicella cases. The system consisted of 200–350 General practices (GP) (consisting of “Praktische Ärzte, Allgemeinärzte and hausärztlich tätige Internisten”) and between 350–500 pediatric practices (mean monthly numbers), depending on the year. Consistent data was collected from the beginning of 2005 to the end of 2017. Participation was voluntary and no financial incentives were provided. By design, the distribution of the sentinel practices resembled the distribution of all GP and pediatric practices throughout the country. Every month, all practices were asked to fill in a paper form with the cumulative number of varicella cases (including zero cases) that met the clinical case definition during the last month, with additional information on patient age (stratified into < 1, 1–4, 5–9, 10–14, 15–19 and >  = 20 years) along with the practice ID. This ID—which served as unique identifier—was linked to a separate database at the RKI which among others gave information on the type of practice (GP vs. pediatric) and the federal state where the practice was based at. Cases were counted as per practice. All practices were outpatient facilities. More detailed information on the Sentinel can be found in the literature [4, 5].

#### Statutory health insurance claims data (*I*)

In Germany, around 85% of the population are members of the statutory health insurance system, the other 15% having private health insurance. The statutory health insurance claims data are generated by all outpatient physicians (irrespective of specialization) that are registered with one of the 17 associations of statutory health insurance physicians (ASHIP) in Germany. There is one ASHIP per federal state, North Rhine-Westphalia being the exception with 2 ASHIP. In order to being reimbursed by the ASHIP, physicians electronically collect information on, among others, patient name, age, date of birth, gender, main diagnosis and diagnostic category for every consultation. This information is then sent electronically to the responsible ASHIP every quarter. The date of the consultation is not provided. This means that in terms of time, consultations can only be allocated to the respective quarter. In general, there are 4 different diagnostic categories that can be linked to a diagnosis, usually reflecting the diagnostic certainty. Those categories are “confirmed”, “suspicion of”, “recovered” and “ruled out” (“Gesichert”, “Verdacht auf”, “Zustand nach”, “Ausschluss”). Every ASHIP sent quarterly relevant patient related data in an anonymized form to a central database at the Robert Koch Institute which forms the National vaccination monitoring project. The project has been described in more detail by Rieck et al. in 2014 [6].

#### Countrywide notification system data (*N*)

Any person with suspected varicella as diagnosed by a physician or any person in whom a laboratory test has confirmed an acute varicella infection should be reported to the local health authorities. Reporting is mandatory according to Sects. 6 and 7 of the German Infection Protection Act. The presence of clinical symptoms that are typical for varicella (usually assessed by a doctor) with or without laboratory or epidemiological confirmation is needed in order to fulfill the reference definition for a varicella case. Thus, the reporting by physicians and laboratories is essential for the detection of varicella cases. All physicians involved with patient care (inpatient and outpatient) are obligated to report within 24 h starting from the initial clinical suspicion.

The countrywide notification system data was retrieved through the national notification database and case information on age (by full years), gender, reporting date (by month/year), reporting federal state and hospitalization were collected. As the countrywide notification of varicella was introduced in March 2013 and data for that year are incomplete, we only include cases from 2014 onwards.

More information on the German countrywide notification system can be found online (in German language) [7].

### Estimation of age-specific varicella incidences

#### Extrapolation of varicella incidence from sentinel data (*S*)

The sentinel data provide the number of cases per month per participating practice. In order to estimate incidences for the whole population, we first calculated a monthly patient/practice index for every federal state. This index displays the number of varicella patients that have been diagnosed on average per sentinel practice in that given month and federal state. The method of using practice indices in order to estimate incidences has been described by Zoch-Lesniak et al. in 2018 [4]. Assuming that the sentinel practices are (in number of patients) representative of all practices in that federal state, we multiplied the patient/practice index with the total number of practices in that federal state. This was done for each practice category (GP vs. pediatric) separately. The information on the number of practices in each federal state per year was provided upon request by the German registry of physicians (“Deutsches Arztregister”). Thus, the total number of cases for each age stratum per month per federal state could be estimated. Subsequently, the estimated number of cases of all federal states were summed up per month, resulting in the estimated total monthly number of cases for the whole country. To calculate incidences, population data from the information system of the Federal Statistical Office were used [8].

As incidence calculation for the Sentinel data was based on the sample of participating physicians over the years, these incidences should rather be considered estimates that are accompanied by a certain degree of uncertainty, in contrast to the incidences derived from total population samples for the notification and insurance claims data.

#### Calculation of varicella incidence from statutory health insurance claims data (*I*)

Varicella diagnosis in this dataset was identified with the respective ICD-10 codes. As many patients with a diagnosis of varicella see several doctors or the same doctor more than once, several observations for each patient were anticipated. For this analysis, we only kept the first observation where a patient´s varicella diagnosis was classified as either confirmed (“gesichert”) or recovered (“Zustand nach”). To calculate incidences, we used data on all members of the statutory health insurance system, stratified by the mentioned age groups. For the age group from 0–14 years, membership numbers are collected for the whole group. No data on more detailed age strata within that group were available. As our calculations depend on the specific number of members for our age strata, we calculated the fraction of the insured aged 0–14 years in relation to the total population aged 0–14 years. Once that fraction was obtained, it was multiplied with the total population in our prespecified age strata (i.e. < 1, 1–4, 5–9, 10–14 years) in order to receive stratum specific number of members.

#### Calculation of varicella incidence using countrywide notification system data (*N*)

Here, no calculation needed to be performed as age-specific incidences are readily available and can be extracted from the national surveillance database [9].

### Data management

For data management and calculation of incidences for Sentinel and notification data, Microsoft Excel Version 2010 and MS Access were used. Incidence calculation of insurance data, creation of tables, graphs and statistical tests were performed in R using RStudio (R Foundation for Statistical Computing and RStudio, Inc). For more adequate comparison with the statutory health insurance claims data, months within each year were stratified into quarters for the countrywide notification system data as well as the sentinel data. For better comparison with the sentinel data, both other datasets were age-stratified equivalent to the sentinel stratification (< 1, 1–4, 5–9, 10–14, 15–19 and >  = 20 years).

### Data protection and ethical clearance

No new information on patients was gathered. All data analyzed had already been collected at the time the study had started. All data used had either already been anonymized before being sent to the RKI (Countrywide notification system data and statutory health insurance claims data) or were collected as anonymized, aggregated data (number of cases per month per practice, by age group) in case of the Sentinel data. The group size of each aggregated data unit was so big that it did not allow for inferences concerning any individual case included. Data for Sentinel and statutory health insurance claims data are not publicly available. Countrywide notification system data are readily available (Link: https://www.rki.de/EN/Content/infections/epidemiology/SurvStat/survstat_node.html). Informed consent and ethical committee clearance had already been attained for the Sentinel and statutory health insurance claims data before those data were collected. The procedure of the sentinel study and the submission of anonymized sentinel data was proved and permitted by the Federal Data Protection Commissioner and proved by the Ethics Committee of Charité Berlin. For the statutory health insurance claims data, the transmission of data for the purpose of analysis over longer periods of time from the health insurance companies to federal and state health reporting institutions like the RKI is legitimized by the Social Security Code V (SGB V), which came into force in 2003.

## Results

**Fig. 1:**
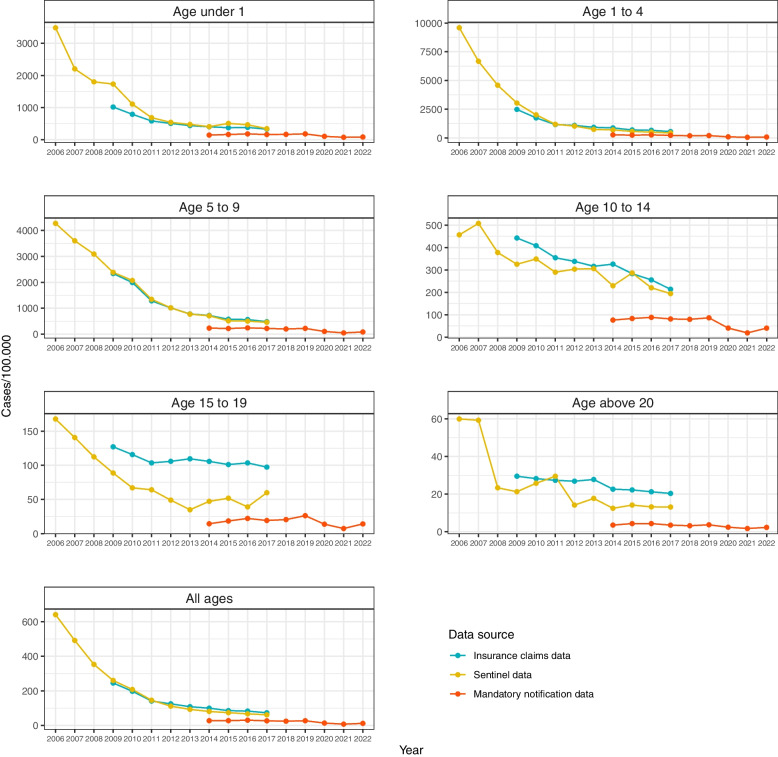
Age-specific varicella incidences (cases/100,000) by year and age group, 2006-2022 , Germany

As can be seen in Fig. 1, both Sentinel and insurance claims data show a clear reduction of overall varicella incidence since the beginning of data collection in 2006 and 2009, respectively. This decrease in case numbers could be observed for all pediatric age groups as well as adults above 20 years, throughout almost all years under observation. All incidences in *N* were constantly below the incidences in *S* an *I* in the years 2014 to 2017.

The age groups up to 9 years which showed the highest incidence at the beginning were the ones with the strongest subsequent absolute and relative reductions, with decreases in case numbers ranging from fourfold to more than 20-fold (Tables 1 and 2). A decrease in incidence could be described in the first years following the introduction of the vaccination recommendation, also in those age groups that were not included in the above recommendation.

**Table 1:** Age-specific varicella incidences (cases/100.000) and relative changes per year, Sentinel data, 2006–2017, Germany

Year	Age group in years													
	Under 1		1 to 4		5 to 9		10 to 14		15 to 19		Above 20		All ages	
	Incidence	Relative change	Incidence	Relative change	Incidence	Relative change	Incidence	Relative change	Incidence	Relative change	Incidence	Relative change	Incidence	Relative change
2006	3483.4	NA	9590.7	NA	4269.8	NA	456.5	NA	167.7	NA	59.9	NA	640.3	NA
2007	2204.7	-36.7%	6669.4	-30.5%	3599.3	-15.7%	507.7	11.2%	140.6	-16.2%	59.2	-1.2%	491.1	-23.3%
2008	1801.7	-18.3%	4584.8	-31.3%	3087.8	-14.2%	377.6	-25.6%	112.3	-20.1%	23.3	-60.6%	352.7	-28.2%
2009	1732.4	-3.8%	3033.2	-33.8%	2383.1	-22.8%	325.4	-13.8%	88.8	-20.9%	21.3	-8.6%	259.9	-26.3%
2010	1109.0	-36%	2003.9	-33.9%	2070.2	-13.1%	348.7	7.2%	67.1	-24.4%	25.7	20.7%	207.8	-20%
2011	686.6	-38.1%	1182.3	-41%	1344.1	-35.1%	289.8	-16.9%	64.2	-4.3%	29.5	14.8%	145.6	-29.9%
2012	540.5	-21.3%	1024.1	-13.4%	1016.0	-24.4%	303.5	4.7%	49.1	-23.5%	14.1	-52.2%	111.4	-23.5%
2013	477.1	-11.7%	740.5	-27.7%	773.2	-23.9%	305.9	0.8%	35.0	-28.7%	17.7	25.5%	92.8	-16.7%
2014	407.5	-14.6%	691.8	-6.6%	709.2	-8.3%	229.3	-25%	47.3	35.1%	12.4	-29.9%	80.7	-13%
2015	508.7	24.8%	561.0	-18.9%	515.7	-27.3%	286.5	24.9%	51.9	9.7%	14.2	14.5%	73.7	-8.7%
2016	466.9	-8.2%	500.7	-10.7%	504.8	-2.1%	220.1	-23.2%	39.2	-24.5%	13.2	-7%	67.1	-9%
2017	344.7	-26.2%	427.4	-14.6%	459.1	-9.1%	194.3	-11.7%	60.0	53.1%	13.1	-0.8%	61.6	-8.2%
Total: 2006 vs 2017	-90.1%		-95.5%		-89.2%		-57.4%		-64.2%		-78.1%		-90.4%

**Table 2:** Age-specific varicella incidences (cases/100.000) and relative changes per year, Insurance claims data, 2009–2017, Germany

Year	Age group in years													
	Under 1		1 to 4		5 to 9		10 to 14		15 to 19		Above 20		All ages	
	Incidence	Relative change	Incidence	Relative change	Incidence	Relative change	Incidence	Relative change	Incidence	Relative change	Incidence	Relative change	Incidence	Relative change
2009	1018.0	NA	2477.3	NA	2336.9	NA	442.5	NA	127.1	NA	29.5	NA	245.1	NA
2010	792.0	-22.2%	1740.0	-29.8%	1991.8	-14.8%	408.1	-7.8%	115.7	-9%	28.2	-4.4%	197.5	-19.4%
2011	584.5	-26.2%	1156.9	-33.5%	1284.3	-35.5%	354.2	-13.2%	103.5	-10.5%	27.3	-3.2%	141.1	-28.6%
2012	508.1	-13.1%	1087.0	-6%	1014.5	-21%	338.0	-4.6%	105.8	2.2%	26.9	-1.5%	124.7	-11.6%
2013	440.1	-13.4%	919.5	-15.4%	779.8	-23.1%	316.2	-6.4%	109.5	3.5%	27.8	3.3%	108.3	-13.2%
2014	402.4	-8.6%	868.3	-5.6%	724.0	-7.2%	325.8	3%	105.7	-3.5%	22.6	-18.7%	99.7	-7.9%
2015	374.6	-6.9%	690.0	-20.5%	576.9	-20.3%	283.3	-13%	101.1	-4.4%	22.2	-1.8%	85.6	-14.1%
2016	384.1	2.5%	660.2	-4.3%	564.9	-2.1%	255.5	-9.8%	103.5	2.4%	21.2	-4.5%	82.9	-3.2%
2017	329.2	-14.3%	546.0	-17.3%	482.6	-14.6%	213.6	-16.4%	97.3	-6%	20.3	-4.2%	73.2	-11.7%
Total: 2009 vs 2017	-67.7%		-78%		-79.3%		-51.7%		-23.4%		-31.2%		-70.1%	

A rise in total varicella incidence in 2015 and 2016 for *N* could not be observed in *S* and *I*. While incidences for *N* undulated during 2017 and 2018, a rise in incidence in all age groups occurred in 2019 (Table 3). It should be noted that all age specific incidences calculated by *N* started and remained below the values that were calculated by sentinel or billing data in the first years of the study period.

**Table 3:** Age-specific varicella incidences (cases/100.000) and relative changes per year, Mandatory notification data, 2014–2022, Germany

Year	Age group in years													
	Under 1		1 to 4		5 to 9		10 to 14		15 to 19		Above 20		All ages	
	Incidence	Relative change	Incidence	Relative change	Incidence	Relative change	Incidence	Relative change	Incidence	Relative change	Incidence	Relative change	Incidence	Relative change
2014	145.3	NA	262.7	NA	234.4	NA	76.0	NA	14.5	NA	3.5	NA	27.4	NA
2015	163.4	12.5%	238.4	-9.3%	221.3	-5.6%	83.2	9.5%	18.6	28.3%	4.3	22.9%	27.6	0.7%
2016	181.5	11.1%	257.8	8.1%	244.2	10.3%	88.2	6%	22.3	19.9%	4.3	0%	30.3	9.8%
2017	162.8	-10.3%	214.8	-16.7%	223.8	-8.4%	81.1	-8%	19.4	-13%	3.5	-18.6%	26.7	-11.9%
2018	166.9	2.5%	183.5	-14.6%	201.4	-10%	79.6	-1.8%	20.6	6.2%	3.2	-8.6%	24.5	-8.2%
2019	184.1	10.3%	195.6	6.6%	220.5	9.5%	86.1	8.2%	26.9	30.6%	3.7	15.6%	27.3	11.4%
2020	105.0	-43.0%	87.88	-55.1%	106.5	-51.7%	40.4	-53.1%	13.9	-48.3%	2.4	-35.1%	13.7	-49.8%
2021	77.0	-26.7%	51.2	-41.7%	51.1	-52.0%	18.9	-53.2%	7.6	-45.3%	1.7	-29.2%	7.6	-44.5%
2022	85.4	10.9%	73.7	43.9%	86.9	70.1%	40.2	112.7%	14.3	88.2%	2.3	35.3%	12.0	57.9%
Total: 2014 vs 2019^a^	25.5%		-24.7%		-4.4%		13.3%		82.1%		5.7%		-0.7%	
Total: 2014 vs 2022^a^	-41.2%		-71.9%		-62.9%		-47.1%		-1.4%		-34.3%		-56.2%	

^a^Separate calculations for 2014–2019 and -2022 to illustrate possible effects of non-pharmaceutical interventions during COVID-19 pandemic (from 2020)

For teenagers from 15 to 19 years of age, incidence decreased initially followed by a stable trend undulating between incidences of 35 to 50/100.000 since 2012 (*S*) and between 97 to 103 since 2011 (*I*), respectively. An increase from 39.2 in 2016 to 60.0 in 2017 in the Sentinel data could not be observed in the other two datasets. While the notification data showed a general increase in case numbers in 2019 for all ages, incidence for the age group from 15 to 19 had the biggest relative increase of 28.2% (From 20.7 in 2018 to 26.4 in 2019) (Tables 1, 2 and 3).

In 2020 and 2021, all age-specific incidences decreased significantly, most likely as a consequence of the introduction of non-pharmaceutical interventions during the COVID-19 pandemic.

## Discussion

As our results show, age-specific varicella incidences in Germany have dropped significantly since the introduction of the general recommendations for a varicella childhood vaccination in 2004 and 2009.

This incidence reduction has been most pronounced in the age groups younger than 10 years that comprised the highest proportion of susceptible individuals and thus disease burden. In this respect, the general recommendations for vaccination have reached the envisaged goal of reducing the then high varicella morbidity in Germany.

Moreover, the drop in incidence was not limited to vaccinated age groups only but also included infants, older children, adolescents and adults, possibly due to less circulation of the virus and thus lower infection pressure in the community. This observation illustrates an additional positive effect of an improving, vaccine-induced herd-immunity, namely a reduced risk of infection for vulnerable population groups that might not be eligible for (catch-up) vaccination themselves (e.g. premature infants, immunocompromised, pregnant women).

The fact that the decline in incidences was most pronounced during the first years after introduction of the general recommendation might be explained by its rapidly improving implementation during that time period: while less than 50% of school-aged children had received the 1^st^ vaccine dose in 2009, this number had risen to over 80% by 2014. Since then, vaccination coverage increased only moderately [2].

The initial rise in overall incidence in *N* during the first two years after varicella had become a notifiable disease might be due to an improving notification behavior by physicians over the years. However, it should be noted that these rises were not proportionally distributed across all age groups and that a rise across all age groups was also observed in 2019, even though incidences had been relatively stable for the two years prior. The development in 2019 thus warrants further close observation.

The rise in incidence in the sentinel data for 15–19-year olds in 2017 could not be observed in the same year or the year thereafter in the other two datasets.

Although we found rising incidences for nearly all age groups in 2019, the age group of the 15 to 19-year olds showed the highest relative rise of more than 28%. Whether this signal represents a true, beginning trend could not be determined as varicella incidences for all age groups dropped significantly in 2020 and 2021, most likely due to nonpharmaceutical interventions in conjunction with the COVID-19 pandemic However, we would like to emphasize that the discussed incidences in this age-group in 2017 (*S*) and 2019 (*N*) were still significantly below those observed in the initial years following the introduction of the vaccine recommendation.

As a consequence, we did not find convincing evidence that a shift of age-specific incidences from younger age groups to teenagers has taken place in Germany or that incidences in older age groups had started to rise.

Our findings match well with the published literature where none of the countries that have introduced a general recommendation for a varicella vaccine has observed subsequent significant rises of age-specific incidences in teenagers and (young) adults. In the US, where a general vaccination recommendation has existed since 1995, a decrease of age-specific disease incidences could be repeatedly shown for all age groups [10, 11]. Similar results were reported by Canada, Italy and Australia where reduced hospitalization rates due to varicella among adults were observed [12–14] following the introduction of varicella vaccination. In Turkey, where a single-dose regimen was introduced in 2013, incidences decreased in children between 6 and 17 years and remained largely unchanged in the population aged > 17 years [15]. A recently published systematic review could neither find any evidence in favor of a shift of disease burden towards older age groups [16].

## Limitations

Calculation of incidences for the insurance claims data only included those 85% of the German population that are members of the statutory health insurance system. We are not aware that age-specific varicella incidences in the 15% of privately insured subjects are significantly different from the rest of the population and even if this was the case, this should not significantly distort trends over time.

Concerning the sentinel data, a central assumption preceding the calculation of total incidence rates from the dataset was that the sentinel practices are representative in terms of patient size and geographic distribution of all practices in the federal states. Both aspects were considered during the sentinel design, but a previous internal evaluation suggested that average practice size of sentinel participants was slightly higher than the countrywide average, possibly leading to an overestimation of case numbers. However, we do not believe that this overly affected trends for age-specific incidences over the years.

A limitation that is common to all datasets is possible misclassification of zoster cases as varicella during the notification or reporting process. Given that age-specific incidences in our datasets constantly drop from younger to older adults, we do not believe that this issue had relevant effects on our results.

As our data show, incidences for *N* consistently were below those for *S* and *I* during those years where data was available for all datasets, suggesting a level of underreporting for *N*. Although such underreporting is known for mandatory reporting systems, we believe that age-specific trends in disease incidence would still be detected by the varicella notification system if they occurred in the future.

## Conclusion

Varicella incidence considerably decreased in all age groups in the first years following the general recommendation of varicella childhood vaccination until a plateau was reached. We did not find a relevant rise in age-specific varicella incidence thereafter, especially not among teenagers. The relative rise in this age group in 2019 illustrates the need for continuous disease monitoring through a well-equipped national surveillance system.

## Data Availability

The datasets used and/or analysed during the current study are available from the corresponding author on reasonable request.
